# Diversity of Improved Diploids and Commercial Triploids from *Musa* spp. via Molecular Markers

**DOI:** 10.3390/cimb46110700

**Published:** 2024-10-22

**Authors:** Juliana Rodrigues Sampaio, Wanderley Diaciso dos Santos Oliveira, Luiz Carlos de Souza Junior, Fernanda dos Santos Nascimento, Ricardo Franco Cunha Moreira, Andresa Priscila de Souza Ramos, Janay Almeida dos Santos-Serejo, Edson Perito Amorim, Renata Darilia Moraes de Jesus, Claudia Fortes Ferreira

**Affiliations:** 1 Department of Agricultural, Environmental and Biological Sciencies, Federal University of Recôncavo da Bahia, Rua Rui Barbosa, 710-Centro, Cruz das Almas 44380-000, BA, Brazil; sampaiorodriguesjuliana@gmail.com (J.R.S.); juniorluiz6666@gmail.com (L.C.d.S.J.); ricardofcm@ufrb.edu.br (R.F.C.M.); renatadarilia@gmail.com (R.D.M.d.J.); 2 Department of Biological Sciences, Feira de Santana State University, Feira de Santana 44036-900, BA, Brazil; diacisowanderley@hotmail.com; 3 Embrapa Mandioca e Fruticultura, Rua Embrapa, s/no, Cruz das Almas 44380-000, BA, Brazil; feel.20@hotmail.com (F.d.S.N.); andresa.ramos@embrapa.br (A.P.d.S.R.); janay.serejo@embrapa.br (J.A.d.S.-S.); edson.amorim@embrapa.br (E.P.A.)

**Keywords:** genotyping, germplasm, variability, bananas, plantains

## Abstract

Banana breeding consists of obtaining diploid, triploid, and tetraploid intra- and interspecific hybrids by conventional breeding methods with the objective of aggregating characteristics of agronomic and commercial interest. Given the narrow genetic base of bananas, Embrapa’s Banana Genetic Breeding Program (BGBP) aims at crosses between improved diploids (ID) (ID × ID) and between improved diploids (ID) and commercial triploids (ID × CTP) and tetraploids (ID × CTT), in order to increase the genetic base and variability in bananas regarding agronomic traits of interest and resistance to main biotic and abiotic factors. These improved diploids are resistant to main fungal diseases such as yellow (YSD) and black Sigatoka (BSD) disease and Fusarium wilt (race 1 and subtropical race 4), the latter being one of the most devastating diseases in bananas. The genetic diversity between 22 improved diploids and seven commercial banana triploids was analyzed using DNA molecular markers. Five IRAP (Inter-Retrotransposon Amplified Polymorphism, 7 ISSR (Inter-Simple Sequence Repeats) and 12 SSR (Simple Sequence Repeat) markers were used. The genetic dissimilarity matrix was based on the Jaccard dissimilarity index; clusters were separated using the UPGMA (Unweighted Pair Group Method With Arithmetic Mean) method and cophenetic correlation of 0.8755. This study of the genetic diversity between improved diploids and commercial triploids, based on the genetic dissimilarity matrix, revealed that the most dissimilar diploids were DM23 and DM15 (74%) and DM16 and DM15 (74%). The smallest genetic distances between the improved diploids and commercial triploids were between TCGN25 and DM17 (50%) and TCN26 and DM17 (50%). The genetic distance matrix also revealed important genotypes to be used in crosses in order to maintain good characteristics in commercial triploids when crossed with improved diploids. The results of our study provide better breeding strategies for one of the largest banana-breeding programs worldwide focused on the development of banana varieties resistant to main biotic and abiotic factors.

## 1. Introduction

The genetic improvement of bananas consists of obtaining intra- and interspecific diploid (2n = 22), triploid (2n = 33), and tetraploid (2n = 44) hybrid types by conventional method, with the objective of aggregating characteristics that confer palatability to the consumer, shorter stature, resistance to diseases, and higher productivity in plants [1,2,3,4]. The possible pathways for genetic breeding in *Musa* spp. focuses on obtaining triploids and tetraploids, mainly by crosses with improved diploids [2,5]. Thus, targeted crosses require the availability of a genetically diverse and structured germplasm bank. Well-characterized accessions to morphoagronomic and molecular criteria enable strategic selection of genitors and management of collections with gene flow monitoring, management of variability between clones, and maintenance of diversity and rare alleles [6,7,8], which can be used in transformation and gene-editing techniques.

Bananas (*Musa* spp.) resulting from intra- or interspecific natural hybridizations, mutations, or breeding programs have their origin in the diploid wild species *Musa acumminata* Colla (AA) and *M. balbisiana* Colla (BB) and can be classified according to their genomic group and ploidy level into diploids (AA, AB), triploids (AAA, AAB, ABB) and tetraploids (AAAA, AAAB, AABB, ABBB) [9,10]. Triploid bananas can be obtained by crosses between diploids and tetraploids, while tetraploids are obtained by crosses between triploids and diploids. They are also classified into subgroups according to fifteen descriptors based on their morphoagronomic characteristics [11]. However, bananas from the ‘Cavendish’ (AAA) and ‘Terra’ (AAB) plantains make up most of the bananas planted worldwide. The narrow genetic base of this important global economic activity highly increases the risk of attacks by pests and facilitates the occurrence of diseases [12].

In Brazil, the main types and varieties cultivated are the diploid ‘Ouro’ (cultivar Ouro—AA) and the triploids ‘Nanica’, ‘Nanicão’, ‘Grande Naine’ (subgroup Cavendish—AAA), ‘Prata’, ‘Pacovan’, ‘Prata Anã’ (subgroup Prata—AAB), ‘Silk’ (Silk-type—AAB), and ‘Terra’ and ‘D’Angola’ (subgroup Terra—AAB) [3,13]. Although these cultivars are predominantly planted, the bananas of the AAB group, characteristically susceptible to diseases, are destined for the domestic market. The ‘Grande Naine’—the AAA genomic group, also susceptible to diseases, contributes to an incipient Brazilian export market, following the world trend [4,14]. Therefore, the development of new varieties is of utmost necessity.

Several characteristics of edible bananas are due to the contribution of the genomes of *Musa acuminata* and *M. balbisiana*. The characteristics derived from genome A originate in the complex of subspecies such as banksii, burmannica, malaccensis, and zebrina, which constitute the species *M. acuminata* [15]. Resistance to diseases, such as yellow Sigatoka, black Sigatoka, and Fusarium wilt, and resistance to pests, such as nematodes, make up the main contributions of the AA germplasm to breeding programs [16]. The importance of the contribution of the B genome of *M. balbisiana* comes from its greater tolerance to biotic and abiotic stress, greater vigor, and the enrichment of its genome with dominant expression of genes related to the ethylene biosynthesis route and starch production and degradation, with a strong influence on fruit ripening and post-harvest qualities [17]. Thus, with all the specific knowledge already accumulated about the genomic constitution of wild species, it is still a challenge to understand the heritability and interaction between the genes involved in defense responses in *Musa* spp, mainly due to the genetic complexity and polyploidy of the genus.

With approximately 400 accessions, the *Musa* spp. germplasm collection at Embrapa is considered one of the largest in the world, harboring accessions collected during expeditions in Southeast Asia, Africa, and Central and South America and many other unique accessions not found elsewhere, such as wild and improved diploids. These improved diploids have been the products of an ongoing breeding program since 1983, where morphoagronomic, physico-chemical, and, recently, molecular characterizations have played an important role. The BGBP at Embrapa uses two strategies to develop new varieties: (i) crosses between improved diploids × improved diploids to create variability and (ii) crosses between improved diploids and commercial triploids. In the first strategy, the aim is to cross genetically distant diploids, and in the second strategy, to cross improved diploids with commercial triploids with a more similar genetic background. This latter strategy seeks to maintain the good agronomic characteristics of the varieties already in the market but with aggregated value, such as disease resistance.

Genetic markers play an important role in studies of diversity and variability because they provide a large amount of data and are not affected by the environment [18,19]. Plant genomes have abundant DNA regions with repetitive sequences, such as transposable elements and microsatellites, which are conserved, frequent, and sometimes common to several species, enabling their use as molecular markers in polymorphism detection. In the *Musa* genus, 41.85% and 55.75% of genomes A and B, respectively, are composed of repetitive elements [17]. Deep genomic studies, mostly based on whole sequencing, provide relevant information about banana plants [15,17,20]. However, traditional tools such as marker systems based on the detection of polymorphism in microsatellites (SSR and ISSR) and retrotransposons (IRAP) are informative and widely used in diversity studies in various species, including genome cultures as complex as the genus *Musa* L. [21,22,23]. In addition to diversity studies, it has been shown that it is possible to identify QLTs related to salinity and drought tolerance in rice and barley with ISSR, SSR, and IRAP markers [24,25,26], as well as the population structure of 240 Argania spinosa accessions using 4 IRAP and 7 ISSR markers [27].

Reproducibility, relatively low cost, and simplicity of execution and analysis are characteristics that, associated with the robustness of the results, confer high potential for the use of IRAP, SSR, and ISSR as tools to support the management of active gene banks and plant breeding and conservation programs in regions with limited access to technological advances in genomic research. Therefore, in this study, IRAP, ISSR, and SSR markers were used to analyze genetic diversity in a collection of 29 *Musa* spp. Genotypes, including improved diploids (AA) and commercial triploids (AAA, AAB), represent the Cavendish subgroup, Prata-type, Silk-type, and plantains. The DNA markers used made it possible to identify potential genitors to be used in the BGBP, given the strategies at play.

## 2. Materials and Methods

### 2.1. Plant Material

The list of 22 improved diploids (AA) and seven commercial triploids (AAA, AAB) from *Musa* spp. is detailed in Table 1. The genotypes evaluated belong to the Banana Germplasm Collection (BGC) at Embrapa Mandioca e Fruticultura, located in the municipality of Cruz das Almas, Bahia, at coordinates 12°40′47.2296″ S, 39°5′17.0952″ W, and 220 m above sea level. The climate of the municipality is hot and humid, classified as tropical (Af), with an average temperature of 23.7 °C, an average annual rainfall of 1.161 mm, and 2327.88 h of annual sunlight [28]. These improved diploids are resistant to main fungal diseases such as yellow (YSD) and black Sigatoka (BSD) disease and Fusarium wilt (race 1 and subtropical race 4), the latter being one of the most devastating diseases in bananas [29,30,31,32,33].

### 2.2. DNA Extraction

Genomic DNA from each genotype was extracted from young and fresh banana leaves (300 mg) following the modified CTAB protocol [34] with maceration of plant material in the presence of 3 mL of extraction buffer, using an adapted bench drill [34]. The quantity and quality of the extracted DNA were evaluated by photodocumentation over UV light after agarose gel electrophoresis (1%) at 70 V for 40 min. Quantification was inferred from visual comparison with reference Lambda DNA with 100, 200, and 300 ng (Invitro Gen 1 Kb ladder) and 100 bp, Promega 1 Kb). Total DNA was diluted in extraction buffer for use in IRAP, ISSR, AND SSR analyses.

### 2.3. IRAP Analysis

PCR-IRAP procedures were performed based on [7,35] with adaptations in amplification programming. Long Terminal Repeat—LTR—retrotransposon-based markers from the Nikikita and Bare 1 families, specifically the sorghum-originated primers (*Hordeum vulgare* L.), Nikita, 5′LTR2, 3′LTR, LTR6150, LTR6149, and C0795 (Table 2), were used in combination for the IRAP analysis. PCR-IRAP and PCR-REMAP amplification reactions were performed with a final volume of 15 µL per sample, containing 4 µL of template DNA (40 ng), 0.75 µL of MgCl_2_ (50 mM), 1.5 µL of 10 × PCR buffer, 1.2 µL of dNTP (2.5 mM), 1.5 µL of each primer (10 mM) paired, 0.3 µL of commercial Taq DNA polymerase (5 U·µL^−1^), and nuclease-free water.

The amplification protocol consisted of programming an initial denaturation step at 94 °C for 3 min, followed by 35 cycles consisting of denaturation at 94 °C for 30 s, annealing temperature (45 °C to 48 °C) for 60 s, and extension at 72 °C for 45 s per cycle, followed by final extension with maintenance at 72 °C for 5 min and completion of the reaction with a reduction in temperature to 10 °C, maintained until the samples were removed from the thermalcycler.

For this work, only the markers that generated electrophoretic profiles with good resolution, with easily visualized and primable bands, were considered. The same procedure was adopted for the other reactions and marker systems. 

### 2.4. SSR Analysis

Twelve SSR markers were used (Table 3), selected from [36] for the analysis. Reactions were performed in a final volume of 20 µL for each sample, containing 1.5 µL of MgCl_2_ (25 mM), 2.0 µL of 10 × PCR buffer, 1.0 µL of dNTP (2.5 mM), 0.8 µL of each primer (5 mM), 0.3 µL of commercial Taq DNA polymerase (5 U·µL^−1^), 3.0 µL of DNA (30 ng·μL^−1^), and nuclease-free water. The amplification program consisted of 2 min of initial denaturation at 95 °C, followed by 39 cycles consisting of 60 s of denaturation at 94 °C, 60 s at annealing temperature (Table 3), followed by 60 s of extension at 72 °C, and 10 min of final extension at 72 °C.

### 2.5. ISSR Analysis

Seven ISSR primers (Table 4) were selected. The PCR reaction mix consisted of a final volume of 25 µL containing 2.5 µL MgCl_2_ (25 mM), 2.5 µL 10× PCR buffer, 2.0 µL dNTP (2.5 mM), 3.5 µL primer (2 mM), 0.2 µL commercial Taq DNA polymerase (5 U·µL^−1^), 4.0 µL DNA (40 ng·µL^−1^), and nuclease-free water. The amplification program consisted of 3 min of initial denaturation at 94 °C, followed by 39 cycles consisting of 40 s of denaturation at 94 °C, 40 s at annealing temperature (Table 4), followed by 60 s of extension at 72 °C and 5 min of final extension at 72 °C.

### 2.6. Electrophoresis and Data Analysis

All products were stained with 3 µL of running buffer solution containing Red^®^ Gel. The amplified segments were separated by 2% agarose gel electrophoresis (IRAP and ISSR) and 3% (SSR) in 5 × TBE buffer, under a constant voltage of 85 V for approximately three hours and were photographed under UV light, using the Loccus L-PIX EX 25 × 30 photodocumentation image-capture system. The length of the amplified bands was inferred by comparison to the reference ladder Invitrogen Plus and PROMEGA 1 Kb (IRAP and ISSR) and Invitrogen Plus 100 bp (SSR). PCR amplification profiles were all scored as dominant markers, adopting (1) for presence and (0) for absence for each polymorphic band position for each primer [18].

The genetic distance matrix, dendrogram, and phenotypic correlation based on 1000 resamples were obtained using the R 4.3.2. software, with packages *prabclus* and *Nbclust* [37]. The principal coordinate analysis (PCoA) was obtained from the R program and the *vegan* package. The UPGMA method and the cutoff point were defined based on the criteria proposed by [38] for cluster formation.

## 3. Results

### 3.1. Molecular Analysis of Gene Polymorphism by DNA Molecular Markers—SSR, ISSR, and IRAP

**Figure 1:**
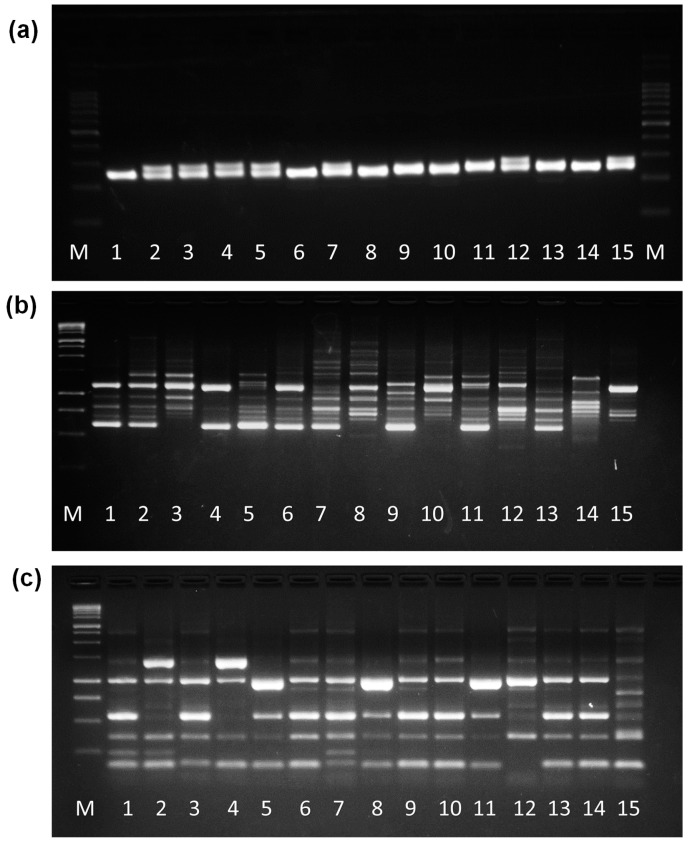
(**a**) Electrophoretic profile of 15 samples (AA-*Musa* spp.) on 3% agarose gel using SSR tag (mMaCIR 8); (**b**) Electrophoretic profile of 15 diploid samples (AA—*Musa* spp.) on 2% agarose gel using ISSR tag (ISSR 95), and (**c**) IRAP LTR6149 + Nikita. M = Ladder marker (Invitrogen^®^, Waltham, MA, USA).

Five reactions were performed with combinations of IRAP primers (3′LTR + C0795, 3′LTR + LTR6150, LTR6149 + Nikita, 5′LTR2 + LTR6150, 5′LTR2 + Nikita), seven reactions with ISSR markers (ISSR 92, 72, 95, 47, 29, 07, 57) and twelve reactions with SSR markers (mMaCIR 01, 07, 28, 24, 40, 150, 45, 196, 231, 13, 39, 152). The representative electrophoretic profile of each type of marker is shown in Figure 1a–c.

For the ISSR markers, 95 total bands with 76 polymorphic bands (80%) were reproduced. Annealing temperatures ranged from 45 °C to 50 °C, with an average temperature of 48.71 °C. The ISSR 07 marker amplified the highest number of polymorphic bands (17 bands) and ISSR 92, the lowest number (2 bands). The size of the amplified bands ranged from 350 to 2500 bp.

IRAP markers generated a total of 60 bands, of which 57 were polymorphic. The average number of bands per primer combination was approximately 12 bands, where the highest and lowest number of polymorphic bands were amplified by the combination of 5′LTR2 + Nikita and 5′LTR2 + LTR6150 primers, 14 and 9 bands, respectively. The annealing temperature ranged from 45 °C to 48 °C, with an average temperature of 45.8 °C. The size of the bands ranged from 2500 bp, amplified by 3′LTR + LTR6150 and LTR6149 + Nikita, to 250 bp, amplified by the 3′LTR + LTR6150, LTR6149 + Nikita and 5′LTR + Nikita marker combinations.

SSR markers amplified a total of 105 bands. The size of the bands ranged from 100 to 400 bp. The annealing temperature ranged from 52 °C to 59 °C, with an average temperature of 55 °C. The highest number of bands was amplified by the mMaCIR 07 marker (14 bands), while the mMaCIR 45 and mMaCIR 152 markers amplified the lowest number of them (5 bands each). The markers mMaCIR 13, mMaCIR 24, and mMaCIR 07 amplified the highest number of polymorphic bands (11 polymorphic bands), while the markers mMaCIR 45 and mMaCIR 152 amplified the lowest number (5 polymorphic bands).

### 3.2. Genetic Diversity

**Figure 2:**
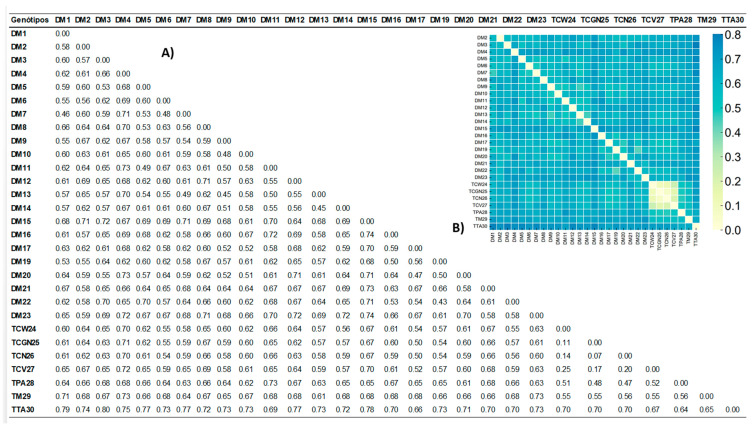
(**A**) Genetic distances based on ISSR + IRAP + SSR data between improved diploids (AA) and commercial triploids (AAA, AAB) of *Musa* spp. belonging to BGC-Banana Germplasm Collection from the PMGB of Embrapa Mandioca e Fruticultura, calculated by complement of the Jaccard dissimilarity Coefficient. (**B**) Illustration—color map of the distances. The identification and details of the genotypes are shown in Table 1.

To increase the clustering power, the combination of the ISSR +IRAP + SSR data sets was chosen for the construction of the dissimilarity matrix based on the complement of the Jaccard dissimilarity Coefficient (Figure 2A,B). The CoPhenetic Correlation Coefficient was 0.8755.

From the analysis of the genetic diversity matrix (Figure 2), the most genetically dissimilar improved diploids were DM23 and DM15 (74%), DM16 and DM15 (74%), DM21 and DM15 (73%), DM20 and DM4 (73%), and DM11 and DM04 (73%). The most genetically similar genotypes between improved diploids and commercial triploids were between TCGN25 and DM17 (50%), TCN26 and DM17 (50%), and TCV27 and DM17 (52%). The average genetic distance between the evaluated genotypes was 58%.

**Figure 3:**
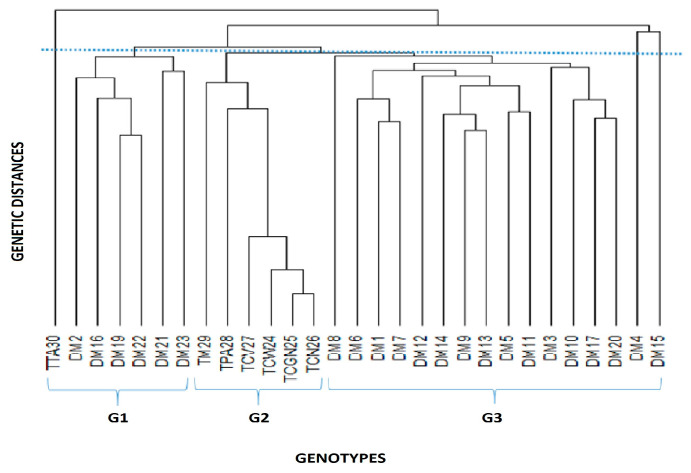
Dendrogram displaying the genetic diversity between 22 improved diploids and 7 commercial triploids of *Musa* spp. separated into three main groups, G1, G2, and G3. The codes of the plants evaluated are shown in Table 1.

Cluster analysis based on the distance matrix grouped genotypes into three groups (Figure 3). The commercial triploid TPA30 (AAB—Plantain, Dwarf Earth), composing an isolated subgroup, and six improved diploids, DM2, DM16, DM19, DM22, and DM23, were clustered in group 1. In group 2, there are all commercial triploids, with the exception of the triploid TPA30. And in group 3, there are all the remaining 16 commercial diploids, DM8, DM6, DM1, DM7, DM12, DM14, DM9, DM13, DM5, DM11, DM3, DM10, DM17, DM20, DM4, and DM15.

**Figure 4:**
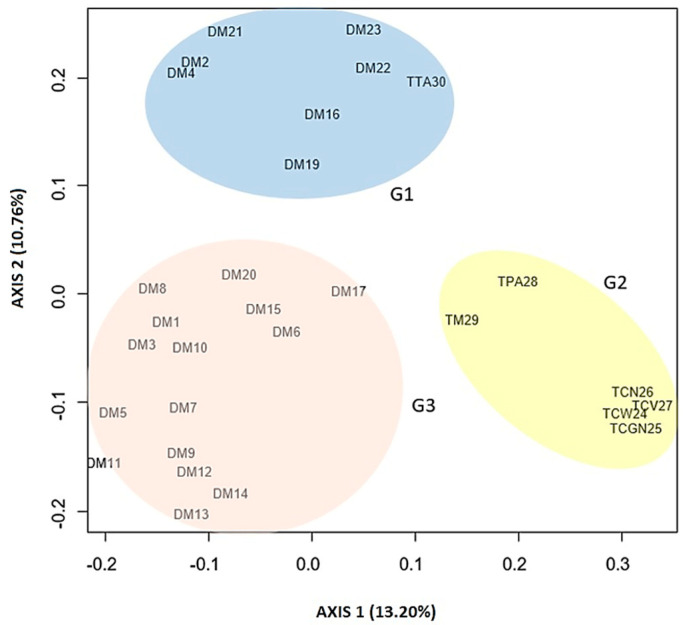
Principal coordinate analysis (PCoA). Twenty-two improved diploids and seven commercial triploids of *Musa* spp. separated into three main groups, G1, G2, and G3. Twenty-four percent of the variability is represented by coordinates 1 and 2. The code of the plants evaluated is shown in Table 1.

The PCoA graph reflects the dispersion of the samples between three groups (Figure 4), as detected in the dendrogram, and presents 24.02% of variability distributed in the first two main coordinates (13.20% in coordinate 1 and 10.76% in coordinate 2).

## 4. Discussion

### Molecular Analysis of Gene Polymorphism and Genetic Diversity

One of the principles that guide most banana-breeding programs is the obtainment of hybrids by conventional crosses [1]. Embrapa’s BGBP continuously aims at crossing diploids to expand the genetic base of *Musa* spp. by generating variability and crossing these improved diploids with triploids and tetraploids for the development of productive, resistant cultivars with the organoleptic characteristics demanded by consumers. Among the main desirable agronomic characteristics associated with *Musa* spp., we have precocity, reduced cycle between harvests, plant height, rusticity, and vigor related to the diameter of the pseudostem and tillers per plant, length, and diameter of the peduncle, and characteristics linked to the productivity and quality of fruits and bunches [30].

The choice of genitors to be used in these crosses is mostly guided by morphoagronomic and molecular characterizations, which are ongoing in the BGBP at Embrapa [39]. In-depth knowledge of the genetic diversity available in the *Musa* spp. collection sets the strategy to guide crosses and accelerate the development of bananas that are more resistant to main biotic and abiotic factors. The diploids that make up this study, as well as their progenitors, were previously evaluated for resistance to black Sigatoka [31,32], Fusarium wilt race 1, and subtropical race 4 [30,33], among other important agronomic [32] and organoleptic characteristics.

IRAP markers amplify centromeric regions between segments consisting of retrotransposon movable elements with LTR long terminal repeats. Retrotransposons are highly capable of increasing genome size (DNA), chromosomal restructurings, and mutations with gene-silencing or overexpression due to their mechanism of self-duplication and insertion into a new locus [7,19]. Microsatellite markers occupy intergenic regions in the genus *Musa* spp., and because they are highly specific markers, they allow for the discrimination of genotypes in diversity studies [40]. Thus, combining the data from different regions of the genome (IRAP, ISSR, and SSR) provides higher clustering power.

From the general analysis of the dissimilarity matrix (Figure 2), it is possible to identify the most dissimilar improved diploids to be crossed among themselves in order to increase the variability and broaden the genetic base. On the other hand, with the aim of maintaining good agronomical qualities of the plant and fruit, the genetically closest improved diploids to triploids are sought out. DM23 (028003-01) and DM16 (042085-02) have 74% dissimilarity in relation to the diploid DM15 (CNPMF 0519). DM23 (028003-01) is a hybrid moderately resistant to black Sigatoka [31] originated from the cross between the cultivar Tuu Gia × the wild subspecies *M. acuminata* spp. *banksii* (‘Madang’). ‘Tuu Gia’ is resistant to yellow and black Sigatoka and *Fusarium oxysporum* f.sp. *cubense* [31,41]. DM16 (042085-02) originates from the cross [M53 × (Madu × Calcuta)]. The DM15-improved diploid (CNPMF 0519) is a wild diploid originated by self-pollination (‘Tambi’ × ‘Tambi’), and although it is completely resistant to black Sigatoka, in the evaluation conducted by Gonçalves et al. [32], it occupied the 17th position in the ranking made by the Mulamba and Mock index based on 18 agronomic traits.

Considering the results obtained by [33], who evaluated 24 improved diploids obtained by the PMGB of Embrapa Mandioca e Fruticultura, the authors identified DM7 (086094-20) as moderately susceptible to Foc ST4, the diploid DM16 (042085-02) as moderately susceptible to Foc R1 and ST4, and DM15 (CNPMF 0519) as susceptible to Foc R1 and ST4; it is possible to prioritize other promising diploids. Thus, DM20 (CNPMF 0557) and DM 11 (013004-04) were identified, with a genetic distance of 73% in relation to DM4 (CNPMF 1323), as the most indicated parents. DM20 and DM4 are resistant to Foc R1 and ST4, while DM11 is highly resistant to Foc R1 and moderately resistant to Foc ST4 [33]. Also, in the evaluation of [32], DM4 and DM20 are potential progenitors because they present resistance to black Sigatoka and the best agronomic indexes.

Aiming at crosses between improved diploids and commercial triploids, the smallest genetic distance between genotypes ranged from 50% to 61% of dissimilarity. The improved diploid DM17 (CNPMF 0536) is the closest to the triploids ‘Grande Naine’ (TCGN25) and ‘Nanica’ (TCN26) with 50% similarity, being 48% similar to the cultivar Valery (TCV27) and 46% similar to the triploid ‘Williams’ (TCW4); all triploids are from the Cavendish subgroup (international export market). It is also the closest to the triploid ‘Terra-Anã’ (TTA30), a plantain.

The diploid DM17 (CNPMF 0536) is a hybrid between [(*Musa acuminata* spp. *burmannica* ‘Calcutta’) × (*Musa acuminata* spp. *banksii* ‘Madang’)] × [(Malaccensis − FHIA × Tjau Lagada)], resistant to Foc R1 and black Sigatoka [30,32]. However, it is important to note that transgenics or induction of mutation and somaclonal variation are other methods that should be encouraged due to the sterility of commercial triploids, given the innate parthenocarpy of these species. Although embryonic sacs at different stages of development are found in triploids of the Cavendish subgroup, Mysore, Plantain, and Prata-type, there is still no consensus on the functionality of these structures in these plants and the sterility hypothesis is still based on incomplete or no growth of the pollen tube or seed degeneration [42]. Recent studies suggest that phenolic components and oxidative enzyme activity in Cavendish negatively impact the growth of pollen tubes [43,44]. Nonetheless, the understanding of infertility as a means of the selection pressure for seedless fruits in banana domestication remains relevant [44] until new research overcomes the barriers that cause sterility in commercial triploids.

The diploid closest to the ‘Prata-Anã’ triploid (TPA28) is DM20 (CNPMF 0557) with 39% similarity. The DM20 originates from [(M61 × Lidi) × (Malaccensis − FHIA × Tjau Lagada)]. In the UPGMA cluster analysis (Figure 3), the triploid ‘Prata-Ana’ (TPA28) and the diploid DM20 (CNPMF 0557) are allocated to groups 2 and 3, respectively. The diploid most similar to the ‘Silk’ triploid (TM29) is DM13 (CNPMF 0542), originated from the [(Malaccensis − FHIA × Sinwobogi) × SH3263 − Hybrid of FHIA) cross. In the dendrogram, the triploid ‘Silk’ and the improved diploid DM13 are allocated in groups 2 and 3, respectively. The improved diploid DM13 (CNPMF 0542) was resistant to Foc race 1 [33] and moderately resistant to black Sigatoka in the evaluation carried out by Gonçalves et al. [32], even under cold environmental conditions, which intensify symptoms in susceptible genotypes.

It is noteworthy to mention that the three improved diploids most similar to commercial triploids presented the FHIA genotype, a Prata cultivar, among their genitors [45]. In the diploids DM17 (CNPMF 0536) and DM20 (CNPMF 0557), the FHIA genotype is integrated as a female genitor, and in the diploid DM13 (CNPMF 0542) as a direct female parent, making up the male parent.

Cluster analysis shows that the set of molecular data ISSR + IRAP+SSR distinguished the improved diploids and commercial triploids, which are allocated in group 2, with the exception of the triploid ‘Terra-Anã’, a plantain. It also represents this trend in the lower dispersion among the triploids of the Cavendish subgroup (AAA) and proximity of the triploids ‘Silk ’and ‘Prata-Anã’ with the exception of the triploid ‘Terra Anã’, which is allocated to group 1, but in an isolated subgroup.

Groups 1 and 3 in the cluster analysis basically consist of improved diploids, with the exception of the triploid ‘Terra-Ana’, a plantain representative of the AAB group forming an isolated subgroup. A larger number of improved diploids are present in group 3 and have the FHIA genotype in the constitution of their genitors. In this group, 10 of the 16 improved diploids have this characteristic. However, DM5 (001016-01), DM6 (M53), DM8 (058054-03), DM12 (050012-02), DM14 (CNPMF 0496), DM15 (CNPMF 0519), and DM20 (CNPMF 0557), are the exceptions.

The value of the cophenetic correlation coefficient was high and adequate (CCC = 0.8755) since CCC > 0.50 is already considered ideal [46], representing a good fit between the representation of the clusters and the data of the genetic distance matrix. The dissimilarity between the diploids is high, ranging from 46% to 74%, which confirms genetic variability. These values are close to the values found by [47] but higher than the values found by [48], using only SSR markers in the molecular characterization of improved diploids. In our work, DNA molecular markers were efficient in demonstrating the genetic variability between the improved diploids and in detecting the similarity between improved diploids and commercial triploids. These findings are key to the BDBP in order to help identify promising crosses to obtain triploids and tetraploids resistant to biotic and abiotic factors in bananas with great agronomic, physico-chemical, and organoleptic characteristics.

The improved diploids were selected for a number of agronomic traits, including the number of fruits per bunch. Therefore, it is expected that their use in crosses will allow for the identification of hybrids superior to the parents (heterosis) in terms of productivity. It is also important to consider that by using improved diploids with more genetic similarity with commercial cultivars, it is possible to better explore the combining ability between parents and the complementarity of genes, which can lead to the development of superior hybrids within the progenies. The selection of diploids with good combining capacity has allowed for the development of seeds and progenies that allow for the selection of superior hybrids and maintain the sensory characteristics close to commercial ‘mothers’ (female genitors). Fruit quality is a major challenge in the progenies generated, more challenging than the identification of disease-resistant hybrids. Even with this limitation, hybrids with sensory quality have been identified in our breeding program, especially from the use of the newly improved diploids, which have greater genetic similarity with commercial cultivars. This work is also important to complement ongoing research at Embrapa focused on sequencing techniques, proteomics, plant × pathogen interactions for identification of differentially expressed genes (DEGs) [49,50,51], and, recently, gene editing via CRISPR-Cas9. As mentioned earlier, Embrapa’s banana genetic breeding program is one of the most renowned programs worldwide, with ongoing crosses, and the work presented here is the first step toward breeding strategies aimed at accelerating the obtainment of better bananas.

Brazil’s banana production is still mostly for consumption. The banana export market does not surpass 1%; however, with markers assisting these crosses, the goal is to accelerate the development of better banana varieties, contributing to fostering the agribusiness of this important fruit and soon providing an increment in the global export market.

## Figures and Tables

**Table 1:** Twenty-two improved diploids (AA) and seven commercial triploids (AAA, AAB) of *Musa* spp., belonging to Banana Germplasm Collection at Embrapa Mandioca e Fruticultura, Cruz das Almas, Bahia.

Ploidy Level	Code	Genotype	Genealogy
**AA**	DM1	CNPMF0612zw	[(M53 × Madu)] × SH3263
**AA**	DM2	CNPMF 0998zw	[(Borneo × Guyod)] × [(Borneo × Guyod) × SH3263]
**AA**	DM3	CNPMF 0534zw	[(Calcutta4 × Madang)] × [(Maccensis-FHIA) × (Tjau Lagada)]
**AA**	DM4	CNPMF 1323zw	[(Malaccensis-FHIA × Sinwobogi)] × [(Calcutta 4 × Madang) × (Borneo × Madang)]
**AA**	DM5	001016-01w	Borneo × Guyod
**AA**	DM6	M53	[(Malaccensis − Kedah × Banksii − Samoa)] × [(Paka × Banksii − Samoa)]
**AA**	DM7	086094-20w	[(Calcutta 4 × Heva)] × SH3263
**AA**	DM8	058054-03w	[(Calcutta 4 × Pahang)] × [(Borneo × Madang)]
**AA**	DM9	013018-01w	Malaccensis-FHIA × Sinwobogi
**AA**	DM10	013019-01w	Malaccensis-FHIA × Tjau Lagada
**AA**	DM11	013004-04w	Malaccensis-FHIA × Madang
**AA**	DM12	050012-02w	Pahang × [(Lidi × SH3263)]
**AA**	DM13	CNPMF 0542zw	[(Malaccensis-FHIA × Sinwobogi)] × SH3263
**AA**	DM14	CNPMF 0496zw	[(M61 × Lidi)] × [(Terrinha × Calcutta 4)]
**AA**	DM15	CNPMF 0519zw	Self (diployd wild Tambi)
**AA**	DM16	042085-02w	[(Madu × Calcutta 4)] × M53
**AA**	DM17	CNPMF 0536zw	[(Calcutta 4 × Madang)] × [(Malaccensis-FHIA × Tjau Lagada)]
**AA**	DM18 *	CNPMF 0357	[(Prata-Anã)] × [(M53 (BGB 06)) × M48]
**AA**	DM19	CNPMF 0731zw	[(Malaccensis-FHIA × Madang)] × [(Tuu Gia × Madang)]
**AA**	DM20	CNPMF 0557zw	[(M61 × Lidi)] × [(Malaccensis-FHIA × Tjau Lagada)]
**AA**	DM21	CNPMF 0513zw	[(M61 × Lidi)] × [(M53 (BGB 06)) × (Kumburgh)]
**AA**	DM22	CNPMF 0993zw	[(Borneo × Guyod) × (Tuu Gia × Madang)] × [Khai × [(Calcutta 4 × Madang)]
**AA**	DM23	028003-01f	Tuu Gia × Madang
**AAA**	TCW24	Williams	Commercial banana varieties
**AAA**	TCGN25	Grande Naine	Commercial banana varieties
**AAA**	TCN26	Nanica	Commercial banana varieties
**AAA**	TCV27	Valery	Commercial banana varieties
**AAB**	TPA28	Prata-Anã	Commercial banana varieties
**AAB**	TM29	Maçã	Commercial banana varieties
**AAB**	TTA30	Terra-Anã	Plantain

* DM18 (CNPMF0357) was not evaluated.

**Table 2:** Details of IRAP primers used in 22 improved diploids (AA) and seven commercial triploids (AAA, AAB) of *Musa* spp., indicating orientation, origin, sequence, and reference authors.

Primers	Family of Origin	Sequence (5′-3′)	Reference
5′LTR2 ←	BARE 1	5′-ATC ATT GCC TCT AGG GCA TAA TTC-3′	[7]
3′LTR →	BARE 1	5′-TGT TTC CCA TGC GAC GTT CCC CAA CA-3′	[7]
Sukkula →	Sukkula	5′-GAT AGG GTC GCA TCT TGG GCG TGA C-3′	[7]
Nikita →	Nikita	5′-CGC ATT TGT TCA AGC CTA AAC C-3′	[7]
LTR6149 →	BARE 1	5′-CTC GCT CGC CCA CTA CAT CAA CCG CGT TTA TT-3′	[7]
LTR6150 ←	BARE 1	5′-CTG GTT CGG CCC ATG TCT ATG TAT CCA CAC ATG TA-3′	[7]
CO795	BARE 1	5′-TCC CAT GCG ACG TTC CCC-3′	[23]
C0945	Sabrina	5′-GCA AGC TTC CGT TTC CGC-3′	[23]

→: Forward; ←: Reverse.

**Table 3:** Details of SSR markers used in the analysis of 22 improved diploids (AA) and seven commercial triploids (AAA, AAB) of *Musa* spp., indicating motif and reference.

Marker	T (°C) *	Motif	Reference
mMaCIR 01	55.0	(GA)20	[36]
mMaCIR 07	53.0	(GA)13	[36]
mMaCIR 08	55.0	(TC)6N24(TC)7	[36]
mMaCIR 24	52.0	(TC)7	[36]
mMaCIR 40	54.0	(GA)13	[36]
mMaCIR 150	54.0	(CA)10	[36]
mMaCIR 45	58.0	(TA)4CA(CTCGA)4	[36]
mMaCIR 196	55.0	(TA)4, (TC)17, (TC)3	[36]
mMaCIR 231	55.0	(TC)10	[36]
mMaCIR 13	57.0	(GA)16N76(GA)8	[36]
mMaCIR 39	52.0	(CA)5GATA(GA)5	[36]
mMaCIR 152	54.0	(CTT)18, (CT)17, (CA)6	[36]

* Annealing temperature in this study.

**Table 4:** Details of ISSR markers used in analyzing 22 improved diploids (AA) and seven commercial triploids (AAA, AAB) from *Musa* L.

Markers	Name	T (°C) *	Sequence	Reference
ISSR 92	TriGAC 3′RC	45.0	GACGACGACGACGACRC	Embrapa
ISSR 72	TriTCC 3′RC	50.0	TCCTCCTCCTCCTCCRC	Embrapa
ISSR 95	TriGTT 3′RC	50.0	GTTGTTGTTGTTGTTRC	Embrapa
ISSR 47	TriTGT5′CY	50.0	CYTGTTGTTGTTGTTGT	Embrapa
ISSR 29	TriCAC3′RC	50.0	CACCACCACCACCACRC	Embrapa
ISSR 07	DiCA5′CY	48.0	CYCACACACACACACACA	Embrapa
ISSR 57	TriACC 3′RC	50.0	ACCACCACCACCACCRC	Embrapa

* Annealing temperature in this study.

## Data Availability

Data can be made available upon request.

## References

[1] Shepherd K., Alves E.J. (1981). The banana breeding programme at the Centro Nacional de Pesquisa de Mandioca e Fruticultura (CNPMF), Bahia State, Brazil. Fruits.

[2] Shepherd K. (1987). Banana Breeding—Past and Present. Acta Hortic..

[3] Silva S.O., Souza Junior M.T., Alves É.J., Silveira J.R.S., Lima M.B. (2001). Banana Breeding Program at Embrapa. Crop Breed. Appl. Biotechnol..

[4] Amorim E.P., Amorim V.B.O., Silva S.O., Pillay M., Pillay M., Tenkouano A. (2011). Quality improvement of cultivated Musa. Banana Breeding: Progress and Challenges.

[5] Creste S., Neto A.T., Silva S.D., Figueira A. (2003). Genetic characterization of banana cultivars (*Musa* spp.) from Brazil using microsatellite markers. Euphytica.

[6] Santos T.T.C., Amorim V.B.O., Santos-Serejo J.A., Silva Ledo C.A., Haddad F., Ferreira C.F., Amorim E.P. (2019). Genetic variability among autotetraploid populations of banana plants derived from wild diploids through chromosome doubling using SSR and molecular markers based on retrotransposons. Mol. Breed..

[7] Teo C.H., Tan S.H., Ho C.L., Faridah Q.Z., Othman Y.R., Heslop-Harrison J.S., Kalendar R., Schulman A.H. (2005). Genome constitution and classification using retrotransposon-based markers in the orphan crop banana. J. Plant Biol..

[8] Saraswathi M.S., Uma S., Prasanya Selvam K., Ramaraj S., Durai P., Mustaffa M.M. (2011). Assessing the robustness of IRAP and RAPD marker systems to study intra-group diversity among Cavendish (AAA) clones of banana. J. Hortic. Sci. Biotechnol..

[9] Simmonds N.W., Shepherd K. (1955). The taxonomy and origins of the cultivated bananas. Bot. J. Linn. Soc..

[10] Häkkinen M. (2013). Reappraisal of sectional taxonomy in *Musa* (*Musaceae*). Taxon.

[11] Baurens F.-C., Martin G., Hervouet C., Salmon F., Yohomé D., Ricci S., Rouard M., Habas R., Lemainque A., Yahiaoui N. (2019). Recombination and Large Structural Variations Shape Interspecific Edible Bananas Genomes. Mol. Bio. Evol..

[12] Ghag S.B., Shekhawat U.K.S., Ganapathi T.R. (2015). Fusarium wilt of banana: Biology, epidemiology and management. Int. J. Pest Manag..

[13] Silva S.O., Amorim E.P., Santos-Serejo J.A., Ferreira C.F., Rodriguez M.A.D. (2013). Melhoramento genético da bananeira: Estratégias e tecnologias disponíveis. Rev. Bras. Frutic..

[14] Centro de Estudos Avançados em Economia Aplicada—CEPEA (2023). Banana. Anuário Hortifruti Brasil: Retrospectiva 2023 & Perspectiva 2024.

[15] Liu X., Arshad R., Wang X., Li W.-M., Zhou Y., Ge X.-J., Huang H.-R. (2023). The phased telomere-to-telomere reference genome of *Musa acuminata*, a main contributor to banana cultivars. Sci. Data.

[16] Silva S.O., Matos A.P., Alves E.J. (1998). Melhoramento genético da bananeira. Pesq. Agropec. Bras..

[17] Wang Z., Miao H., Liu J., Xu B., Yao X., Xu C., Zhao S., Fang X., Jia C., Wang J. (2019). *Musa balbisiana* genome reveals subgenome evolution and functional divergence. Nat. Plants.

[18] Arvas Y.E., Marakli S., Kaya Y., Kalendar R. (2023). The power of retrotransposons in high-throughput genotyping and sequencing. Front. Plant Sci..

[19] Kalendar R., Flavell A.J., Ellis T.H.N., Sjakste T., Moisy C., Schulman A.H. (2011). Analysis of plant diversity with retrotransposon-based molecular markers. Heredity.

[20] Chabannes M., Gabriel M., Aksa A., Galzi S., Dufayard J.F., Iskra-Caruana M.L., Muller E. (2021). Badnaviruses and banana genomes: A long association sheds light on *Musa* phylogeny and origin. Mol. Plant Pathol..

[21] Biswas M.K., Bagchi M., Biswas D., Harikrishna J.A., Liu Y., Li C., Sheng O., Mayer C., Yi G., Deng G. (2020). Genome-Wide Novel Genic Microsatellite Marker Resource Development and Validation for Genetic Diversity and Population Structure Analysis of Banana. Genes.

[22] Hinge V.R., Shaikh I.M., Chavhan R.L., Deshmukh A.S., Shelake R.M., Ghuge S.A., Dethe A.M., Suprasanna P., Kadam U.S. (2022). Assessment of genetic diversity and volatile content of commercially grown banana (*Musa* spp.) cultivars. Sci. Rep..

[23] Wen S., Zhao H., Zhang M., Qiao G., Shen X. (2023). IRAPs in Combination with Highly Informative ISSRs Confer Effective Potentials for Genetic Diversity and Fidelity Assessment in Rhododendron. Int. J. Mol. Sci..

[24] Noryan M., Hervan I.M., Sabouri H., Kojouri F.D., Mastinu A. (2021). Drought Resistance Loci in Recombinant Lines of Iranian Oryza sativa L. in Germination Stage. BioTech.

[25] Razi S.M., Shirzadian-Khorramabad R., Sabouri H., Rabiei B., Moghadam H.H. (2022). Identification of Quantitative Trait Loci Related to Salt Tolerance of Indica Rice RIL Population in Different Growth Stages. Russ. J. Genet..

[26] Makhtoum S., Sabouri H., Gholizadeh A., Ahangar L., Katouzi M. (2022). QTLs Controlling Physiological and Morphological Traits of Barley (*Hordeum vulgare* L.) Seedlings under Salinity, Drought, and Normal Conditions. BioTech.

[27] Pakhrou O., Medraoui L., Yatrib C., Alami M., Filali-Maltouf A., Belkadi B. (2017). Assessment of genetic diversity and population structure of an endemic Moroccan tree (*Argania spinosa* L.) based in IRAP and ISSR markers and implications for conservation. Physiol. Mol. Biol. Plants.

[28] AGRITEMPO Agritempo: Sistema de Monitoramento Agrometeorológico. https://www.agritempo.gov.br/en/.

[29] Amorim E.P., Santos-Serejo J.A., Amorim V.B.O., Ferreira C.F., Silva S. (2013). Banana breeding at Embrapa cassava and fruits. Acta Hortic..

[30] Gonçalves Z.S., Haddad F., de Oliveira Amorim V.B., Ferreira C.F., de Oliveira S.A.S., Amorim E.P. (2019). Agronomic characterization and identification of banana genotypes resistant to Fusarium wilt race 1. Eur. J. Plant Pathol..

[31] Nascimento F.d.S., Sousa Y.M., Rocha A.d.J., Ferreira C.F., Haddad F., Amorim E.P. (2020). Sources of black Sigatoka resistance in wild banana diploids. Rev. Bras. Frutic..

[32] Gonçalves Z.S., de Jesus Rocha A., Haddad F., de Oliveira Amorim V.B., Ferreira C.F., Amorim E.P. (2021). Selection of Diploid and Tetraploid Banana Hybrids Resistant to Pseudocercospora fijiensis. Agronomy.

[33] Santana W.S., Rocha A.D.J., Da Silva W.B., Amorim V.B.O.D., Ramos A.P.D.S., Haddad F., Amorim E.P. (2024). Selection of Improved Banana Diploid Resistant to *Fusarium oxysporum* f. sp. *cubense* Races 1 and Subtropical 4. Agronomy.

[34] Ferreira C.F., Gutierrez D.L., Kreuze J.F., Iskra-Caruana M.L., Chabannes M., Barbosa A.C.O., Santos T.A., Silva A.G.S., Santos R.M.F., Amorim E.P. (2019). Rapid plant DNA and RNA extraction protocol using a bench drill. Genet. Mol. Res..

[35] Baumel A., Ainouche M., Kalendar R., Schulman A.H. (2002). Retrotransposons and genomic stability in populations of the young allopolyploide species *Spartina anglica* C.E. Hubbard (Poaceae). Mol. Biol. Evol..

[36] Christelová P., Valárik M., Hřibová E., Houwe I.V.D., Channelière S., Roux N., Doležel J. (2011). A platform for efficient genotyping in *Musa* using microsatellite markers. AoB Plants.

[37] R: A Language and Environment for Statistical Computing; R Foundation for Statistical Computing: Vienna, Austria, 2015. https://www.R-project.org/.

[38] Mingoti S.A. (2005). Análise de Dados Através de Métodos de Estatística Multivariada: Uma Abordagem Aplicada.

[39] Bered F., Barbosa Neto J.F., Rocha B.M., Pegoraro D.G., Vacaro E., Carvalho F.I.F. (2002). Characterization of wheat germplasm through the cycle and height adaptive characters. Pesq. Agropec. Bras..

[40] Biswas M.K., Biswas D., Yi G., Deng G. (2024). The Musa Marker Database: A Comprehensive Genomic Resource for the Improvement of the Musaceae Family. Agronomy.

[41] Dantas J.L.L., Shepherd K., Soares Filho W.S., Cordeiro Z.J.M., Silva S.O., Alves E.J., Souza A.S., Oliveira M.A. (1993). Programa de Melhoramento Genético da Bananeira em Execução no CNPMF/Embrapa.

[42] Shepherd K., Dantas J.L.L., Goutant-Bakry M.-E., Dzoyem C.U.D., Bakry F. (2023). Development and functioning of the embryo sac in four triploid banana cultivars. Pesq. Agropec. Bras..

[43] Silva M.S., Goes N.H., Santos-Serejo J.A., Ferreira C.F., Amorim E.P. (2021). Phenolic compounds and oxidative enzymes involved in female fertility in banana plants of the Cavendish subgroup. Plants.

[44] Silva M.S., Santana A.N., Santos-Serejo J.A., Ferreira C.F., Amorim E.P. (2022). Morphoanatomy and histochemistry of septal nectaries related to female fertility in banana plants of the ‘Cavendish’ subgroup. Plants.

[45] Donato S.L.R., Arantes A.M., Silva S.O.E., Cordeiro Z.J.M. (2009). Phytotechnical behavior of ‘Prata-Anã’ banana and progeny hybrids. Pesq. Agropec. Bras..

[46] Vaz Patto M.C., Satovic Z., Pêgo S., Fevereiro P. (2004). Assessing the genetic diversity of Portuguese maize germoplasm using microsatellite markers. Euphytica.

[47] Amorim E.P., Lessa L.S., Ledo C.A.S., Amorim V.B.O., Reis R.V., Santos-Serejo J.A., Silva S.O. (2009). Agronomic and molecular characterization of diploid improved banana genotypes. Rev. Bras. Frutic..

[48] Amorim E.P., Reis R.V., Santos-Serejo J.A., Amorim V.B.O., Silva S.O. (2008). Genetic variability estimated in banana diploids through microsatellite markers. Pesq. Agropec. Bras..

[49] Mattos-Moreira L., Ferreira C.F., Amorim E.P., Pirovani C.P., Filho M.A.C., Ledo C.A.S. (2018). Diferentially expressed proteins associated with drought tolerance in bananas (*Musa* spp.). Acta Physiol. Plant..

[50] Rocha A.J., Soares J.M.S., Nascimento F.S., Rocha A.S., Amorim V.B.O., Ramos A.P.S., Ferreira C.F., Haddad F., Amorim E.P. (2022). Molecular, Histological and Histochemical Responses of Banana Cultivars Challenged with *Fusarium oxysporum* f. sp. *cubense* with Different Levels of Virulence. Plants.

[51] Nascimento F.S., Mascarenhas M.S., Boaventura S.C., Souza C.C.H., Ramos A.P.S., Rocha A.J., Soaress J.M.S., Diniz E.E.C., Mendes T.A.O., Ferreira C.F. (2024). Phytoene Desaturase (PDS) Gene-Derived Markers Identify “A” and “B” Genomes in Banana (*Musa* spp.). Horticulturae.

